# Supplementary material for: High-throughput fetal fraction amplification increases analytical performance of noninvasive prenatal screening
Source: Genet Med. 2020 Nov 15;23(3):443–50. doi: 10.1038/s41436-020-01009-5 (PMC7935715; doi:10.1038/s41436-020-01009-5)
Supplement: Supplementary file 1 — supplementary figures_and_tables [file 41436_2020_1009_MOESM1_ESM.pdf]

SUPPLEMENT

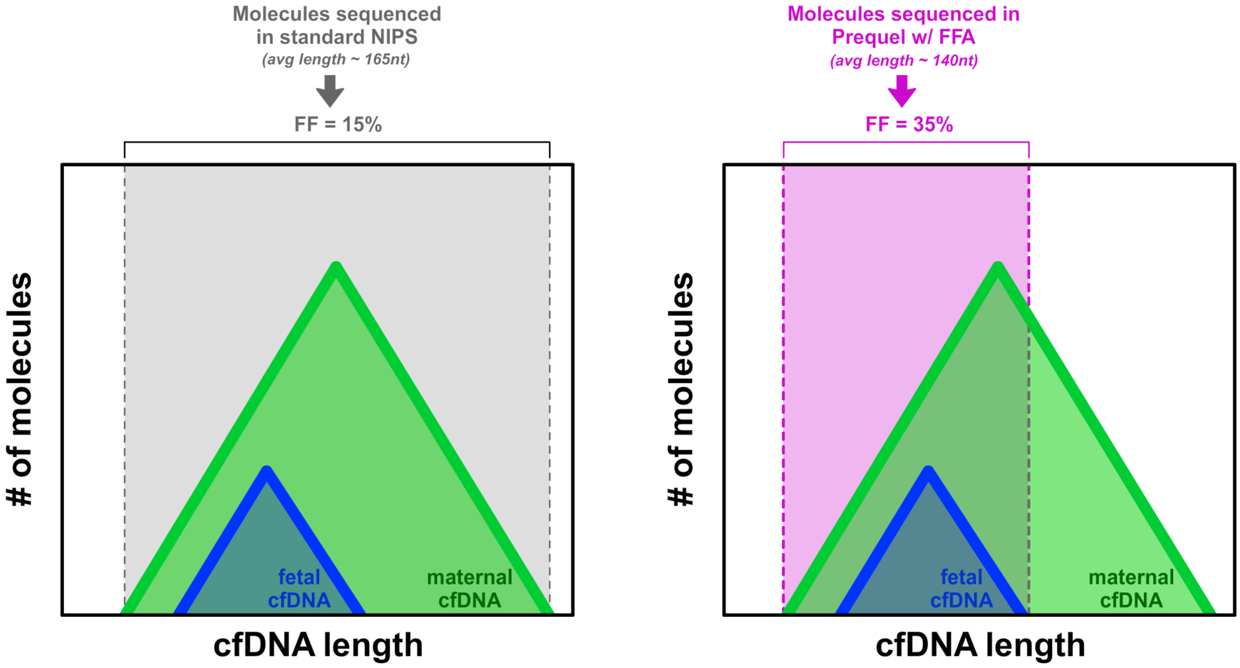

Figure S1: FFA increases the relative concentration of fetal-derived cfDNA fragments via size selection. This schematic example reflects that fetal-derived cfDNA (blue distribution) is both less abundant and systematically shorter than maternal-derived cfDNA (green distribution). Standard NIPS approaches sequence a sampling of all cfDNA, irrespective of length (gray region, left). Using agarose gel electrophoresis to select shorter cfDNA fragments (pink region, right), FFA increases FF because relatively more of the fetal-derived cfDNA distribution is retained as compared to the maternal-derived cfDNA distribution.

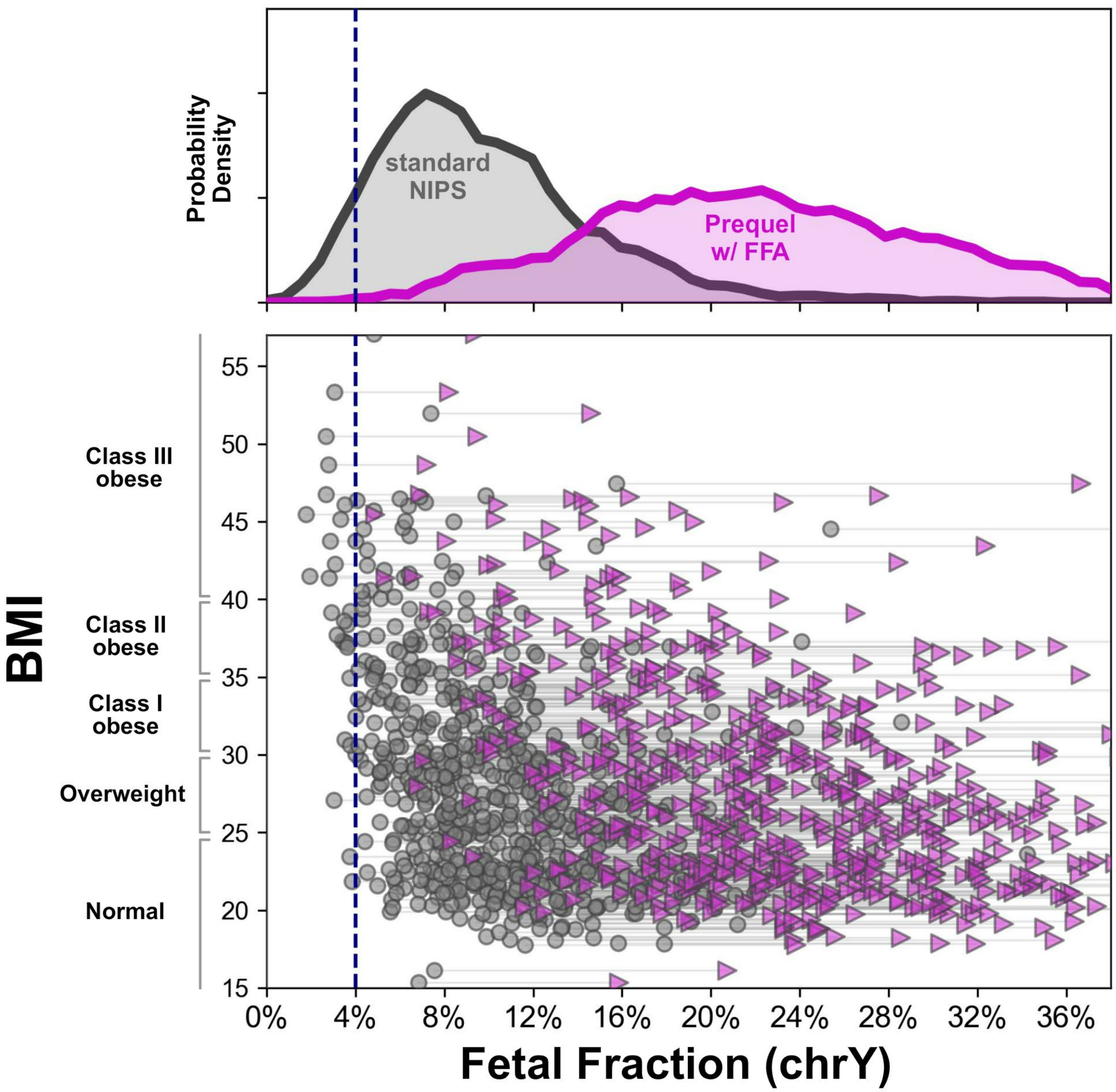

Figure S2: **FFA directly increases the concentration of fetal-derived cfDNA in samples undergoing NIPS.** To illustrate that the results shown in Figure 1 are not the result of analytical artifacts (e.g., in the FF regression from autosomal bin counts applied to male- and female-fetus pregnancies), the FF as measured by the abundance of sequenced cfDNA fragments from chrY is plotted for male-fetus pregnancies.

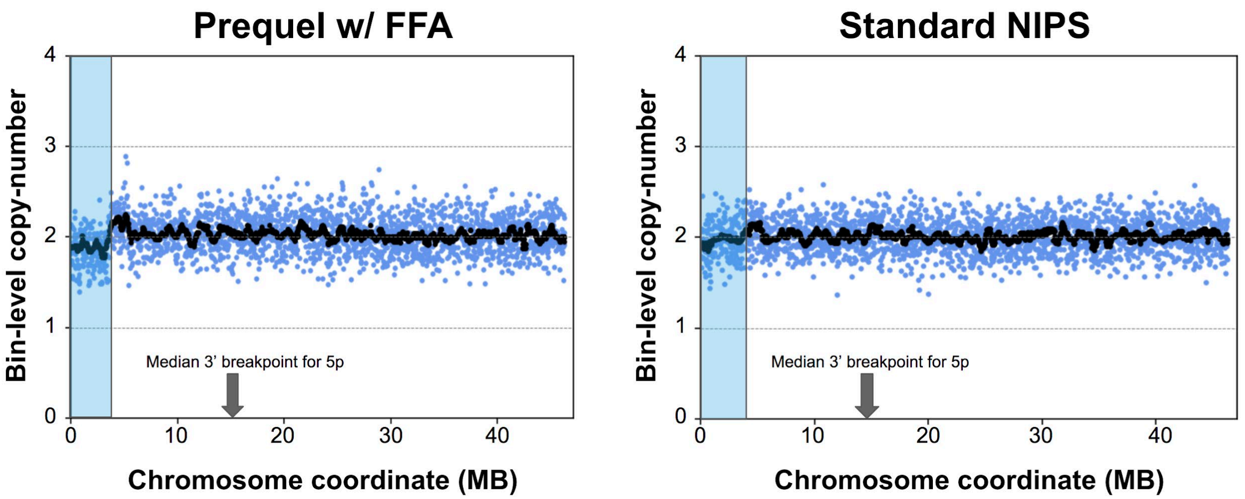

Figure S3: The enhanced fetal fraction of FFA (left) revealed a short microdeletion (~3MB; shaded in blue at left of each plot) in a sample that screened negative with standard NIPS (right). Blue scatter points are the bin-level normalized depth; black scatter points show the rolling median of blue points (median over 25 bin window). This particular deletion is shorter than a typical 5p deletion, for which the median 3' breakpoint is indicated (http://dbsearch.clinicalgenome.org/search/). The FF was 11% with FFA and 7% with standard NIPS.

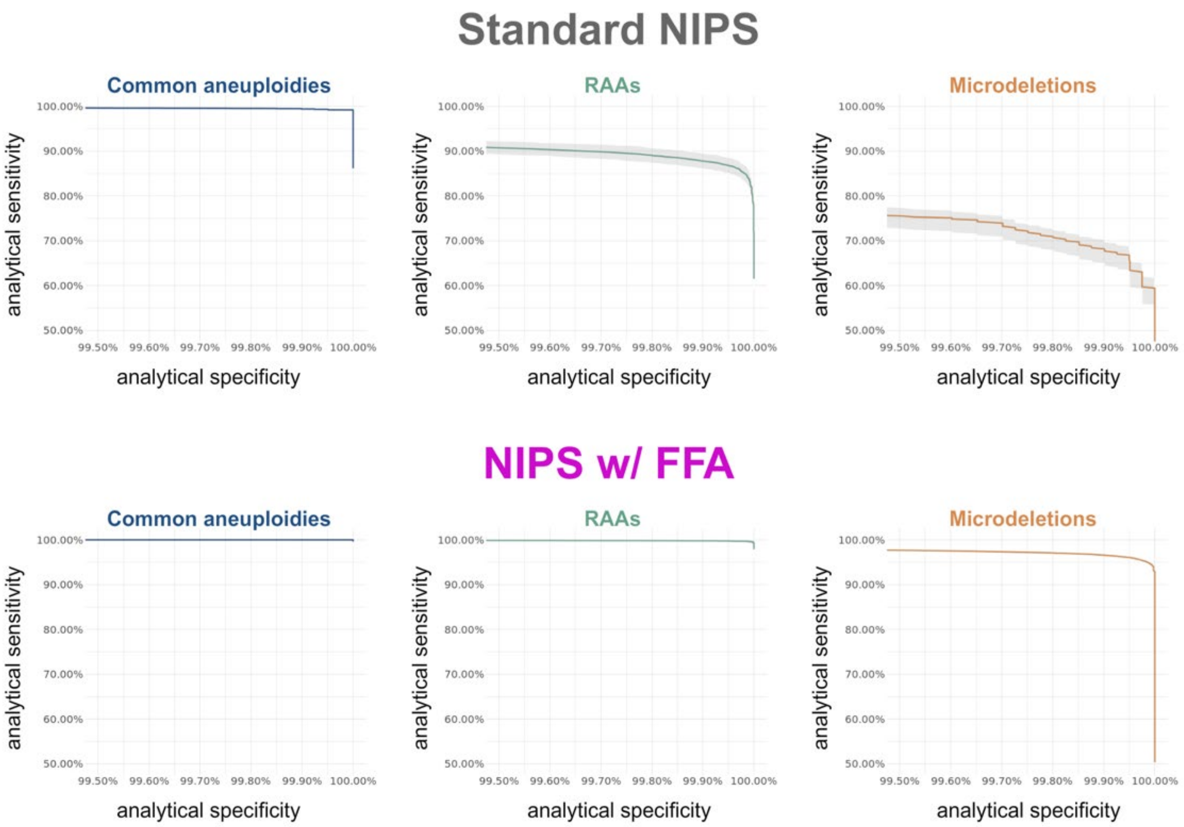

Figure S4: Relative to standard NIPS (top), application of FFA (bottom) increases the analytical sensitivity and analytical specificity of common aneuploidies (left), RAAs (middle), and microdeletions (right).

Aneuploidy	# confirmed positive	FFA clinical sensitivity	FFA clinical specificity
T21	24	100% (CI: 90.2% -100%)	100% (CI: 94.5% -100%)
T18	24	100% (CI: 90.2% -100%)	100% (CI: 94.5% -100%)
T13	10	100% (CI: 78.3% -100%)	100% (CI: 95.8% -100%)
Microdeletion	2	100% (CI: 33.3% -100%)	100% (CI: 96.3% -100%)
SCA	7	100% (CI: 70.8% -100%)	100% (CI: 96.0% -100%)

Table S1: Prequel with FFA had perfect concordance relative to orthogonally confirmed outcome. Sixty-seven samples had confirmed outcome via diagnostic procedure or assessment at birth. One confirmed negative sample was included in the analysis and contributes to the specificity calculation. 95% confidence intervals (CI) were calculated with the Jeffreys-interval approach.

		Replicate 1	
		Negative	Positive
Replicate 2	Negative	50	0
	Positive	0	6

Table S2: Intra-run repeatability. Six screen-positive samples (two each for T13, T18, and T21) and 50 screen-negative samples were tested in duplicate on a single flowcell. The observed percent agreement was 100%.

		Replicate 1	
		Negative	Positive
Replicate 2	Negative	66	0
	Positive	0	9

Table S3: Inter-run reproducibility. Nine samples that were screen-positive for common aneuploidies (three each for T13, T18, and T21) and 66 screen-negative samples were tested in duplicate on separate flowcells. The observed percent agreement was 100%.

		Standard NIPS		Prequel w/ FFA	
	Approximate Prevalence	FNR	Approximate FN / sample screened	FNR	Approximate FN / sample screened
Common aneuploidies	1 in 100	1 in 183	1 in 18,300	1 in 8,333	1 in 833,300
RAAs	1 in 200	1 in 7	1 in 1,400	1 in 344	1 in 66,800
Common microdeletions	1 in 1,000	1 in 4	1 in 4,000	1 in 36	1 in 36,000
DiGeorge Syndrome (22q11.2)	1 in 2,000	1 in 3	1 in 6,000	1 in 22.9	1 in 45,800

Table S4: Estimated # of false negative (FN) results per sample screened with standard NIPS or Prequel with FFA. The false negative rate (FNR) is calculated as (1 - sensitivity), where sensitivity is the analytical sensitivity estimated from the ROC analysis (see Methods). The # of FN per sample screened is the product of the FNR and the prevalence. Prevalence numbers can range based on age and other factors, thus prevalence values are approximate, expressed as 1 in *x*, where *x* is rounded to nearest hundred (for common aneuploidies and RAAs) or the nearest 1000 (for common microdeletions and 22q11.2). The five common microdeletions are 1p, 4p, 5p, 15q11, and 22q11.2.
